# The frequency and duration of *Salmonella*–macrophage adhesion events determines infection efficiency

**DOI:** 10.1098/rstb.2014.0033

**Published:** 2015-02-05

**Authors:** Sarra Achouri, John A. Wright, Lewis Evans, Charlotte Macleod, Gillian Fraser, Pietro Cicuta, Clare E. Bryant

**Affiliations:** 1 Department of Physics, Cavendish Laboratory, University of Cambridge, J J Thomson Avenue, Cambridge CB3 0HE, UK; 2 Department of Veterinary Medicine, University of Cambridge, Madingley Road, Cambridge CB3 0ES, UK; 3 Department of Pathology, University of Cambridge, Tennis Court Road, Cambridge CB2 1QP, UK

**Keywords:** macrophage, salmonella, phagocytosis, flagella, motility

## Abstract

*Salmonella enterica* causes a range of important diseases in humans and a in a variety of animal species. The ability of bacteria to adhere to, invade and survive within host cells plays an important role in the pathogenesis of *Salmonella* infections. In systemic salmonellosis, macrophages constitute a niche for the proliferation of bacteria within the host organism. *Salmonella enterica* serovar Typhimurium is flagellated and the frequency with which this bacterium collides with a cell is important for infection efficiency. We investigated how bacterial motility affects infection efficiency, using a combination of population-level macrophage infection experiments and direct imaging of single-cell infection events, comparing wild-type and motility mutants. Non-motile and aflagellate bacterial strains, in contrast to wild-type bacteria, collide less frequently with macrophages, are in contact with the cell for less time and infect less frequently. Run-biased *Salmonella* also collide less frequently with macrophages but maintain contact with macrophages for a longer period of time than wild-type strains and infect the cells more readily. Our results suggest that uptake of *S.* Typhimurium by macrophages is dependent upon the duration of contact time of the bacterium with the cell, in addition to the frequency with which the bacteria collide with the cell.

## 1. Introduction

*Salmonella enterica* infection is a major cause of disease in humans and animals. Human typhoid fever is responsible for around 27 million cases each year [1,2] and non-typhoidal *Salmonella* (NTS) causes around 93.8 million cases annually [3]. The ability of *Salmonella* to adhere to, invade and survive within host cells is a key feature of infection. Bacterial mutants that are unable to invade or persist within cultured epithelial cells or macrophages exhibit markedly reduced virulence in various animal models [4–8]. Invasion of intestinal macrophages by *Salmonella*, via a process that is dependent on phagocytosis, facilitates their survival, replication and spread throughout the host [8–11].

*Salmonella* are highly motile bacteria and are able to propel themselves through liquid environments by means of several flagella. Each flagellum is a complex structure consisting of a motor driven by proton-motive force, a universal joint and a propeller. The flagellum can rotate clockwise or counterclockwise at angular frequencies in the region of 100 Hz. The synchronicity of flagellar rotation determines two motility regimes: runs and tumbles. During a run all flagella rotate counterclockwise; they are organized in a bundle at one pole of the bacterial cell, which propels it forward. During a tumble flagella rotate clockwise, the bundle disassembles, resulting in tumbling and subsequent reorientation of the bacterium [12]. This bimodal process produces random movement, with bacteria changing direction approximately once a second in uniform environments. This process is biased in response to chemotactic stimuli [13]. Flagellar function is associated with *Salmonella* virulence *in vivo* [14,15].

Motility critically affects the efficiency of how *Salmonella* and other motile bacteria are taken up by macrophages. Non-motile mutants of *Salmonella enterica* serovar Typhimurium exhibit reduced uptake into host cells. *S*. Typhimurium *motA* and *motB* mutants, for example, which have paralysed flagella, are taken up less effectively than wild-type bacteria [16,17]. Mutations in specific chemotaxis genes which bias the rotation of the flagellar motor towards a unidirectional swimming phenotype are hyperinvasive, whereas mutations predisposing the bacterium towards tumbling exhibit substantially reduced invasion of epithelial cells [17]. Loss of bacterial motility is therefore considered an important mechanism by which flagellated Gram-negative bacteria resist uptake by host cells [18].

A number of hypotheses have been suggested to explain the reduced cellular uptake of non-motile bacteria, including that the inability to swim decreases bacterium–cell contact frequency and thus infection efficiency. Consistent with this, centrifugation to increase contact between bacteria and macrophages restores uptake of a *S*. Typhimurium *motA* mutant by macrophages to wild-type levels [16]. Phagocyte uptake of non-motile *Pseudomonas aeruginosa*, *Vibrio cholerae* and *Escherichia coli* mutants is also reduced compared with wild-type strains, but it has been suggested that decreased bacterium–cell contact cannot fully explain this, as centrifugation does not equalize uptake of wild-type and non-motile *P. aeruginosa. Pseudomonas aeruginosa* mutants lacking the *motAB* or *motCD* genes exhibit progressively decreasing motility, which correlates with decreased infection efficiency. A decline in infection is also observed when the activity of the *V. cholerae* flagellar motor is reduced, leading to the hypothesis that the progressive loss of flagellar torsion leads to decreased sensing of the bacteria by phagocytes [18] rather than being a process totally dependent on the frequency of bacterium–cell contact [19].

Our previous work on *Salmonella*–single macrophage interactions in cell-culture conditions has shown heterogeneity in macrophage infection events, such that only a proportion of cells is susceptible to infection [20]. Surprisingly, despite a large number of contacts between bacteria and macrophages, only a very few of these events lead to successful cellular infection [20].

In this work, we investigate the *Salmonella*–macrophage relationship at the single-cell level. We characterize the adhesion and infection dynamics, using live imaging to directly visualize individual cell–bacterial interactions to determine how motility affects the contact between bacteria and macrophages, and their subsequent uptake. Using a panel of *S*. Typhimurium mutants with a range of altered motility phenotypes, we directly compare live single-cell bacterial invasion studies with cellular population analysis. Direct imaging of single-cell infection events shows that contact frequency and duration, and subsequent adhesion are both critical for successful cellular infection to occur.

## 2. Material and methods

### (a) Bacterial strains, cell lines and culture conditions

The bacterial strains and plasmids used in this study are listed in electronic supplementary material, table S1. *S.* Typhimurium was grown on Luria-Bertani (LB) agar or in LB broth, supplemented with 50 µg ml^−1^ kanamycin and 10 µg ml^−1^ chloramphenicol where appropriate, at 37°C under aerobic conditions.

RAW264.7 cells were routinely cultured in Dulbecco's Modified Eagle Medium (DMEM; Sigma-Aldrich) supplemented with 2 mM l-glutamine (Sigma-Aldrich) and 10% fetal bovine serum (PAA Laboratories; hereafter referred to as complete RAW medium) at 37°C, 5% CO_2_.

### (b) Construction of mutant strains

Wild-type *S.* Typhimurium SJW1103 is motile; derived mutants carry stable lesions in flagellar genes *FliOPQR* (SJW192) [21], *ΔmotAB::km^R^*, and *ΔfliM::km^R^* (this study) in which flagellar genes were replaced by a kanamycin resistance cassette, using the *λ* Red recombinase system. Briefly, linear DNA constructs containing kanamycin resistance marker flanked by FRT sites and juxtapose homologous regions to the target deletion gene were generated by PCR using template plasmid pKD13 specific primers (electronic supplementary material, tables S1 and S2). DNA constructs were transformed into wild-type *S.* Typhimurium harbouring pKD46 and selected for incorporation of the kanamycin cassette, and confirmed by PCR. Motility defects could be rescued by *in trans* complementation.

### (c) Construction of expression vectors

Recombinant proteins were expressed from pACTrc derived plasmids (electronic supplementary material, table S1) [22]. To construct recombinant plasmids encoding wild-type and mutated derivatives (see electronic supplementary material, tables S1 and S2 for constructs and oligonucleotides), *S.* Typhimurium *fliM* and *fliN* were amplified from chromosomal DNA by PCR using *Pfu* Ultra DNA polymerase. PCR products were inserted using BamHI-XbaI into pACTrc. Inserts were verified by DNA sequencing.

### (d) Macrophage uptake assays

RAW264.7 cells were seeded at a density of 6 × 10^4^ cells well^−1^ in a 96-well plate in 200 µl complete RAW medium and incubated at 37°C, 5% CO_2_ for 18 h. Three colonies of each *S*. Typhimurium strain were inoculated into 10 ml LB broth with appropriate antibiotics and incubated for 16 h at 37°C, 200 r.p.m. This culture was used to inoculate 10 ml LB broth to a calculated OD_595_ of 0.01, and cultures were incubated at 37°C, 200 r.p.m. for 3–4 h until an OD_595_ of 0.8–0.9 was reached. Cultures were then centrifuged at 3000*g* for 10 min and resuspended in complete RAW medium to 3 × 10^6^ colony forming units (cfu) ml^−1^. Culture medium was removed from RAW264.7 cells and replaced with 200 µl of this bacterial inoculum per well, to give a multiplicity of infection (MOI) of 10 : 1. This MOI was used so that the frequency of infection was high enough to enable observation of infection events at the single-cell level in subsequent imaging experiments, while minimizing *Salmonella*-induced macrophage death [20,23]. The cells were then either incubated at 37°C, 5% CO_2_ for 30 min, or centrifuged for 10 min at 500*g*, followed by incubation at 37°C, 5% CO_2_ for 20 min. The supernatant was removed from each well and 200 µl complete RAW medium containing 50 µg ml^−1^ gentamicin was added; plates were incubated for 1 h at 37°C, 5% CO_2_. The cells were then washed twice with 200 µl DMEM and lysed in 0.5% Triton X-100, and the number of intracellular bacteria (in cfu) was determined by serial dilution and plating on LB agar. Host cell viability was determined using the CytoTox 96 Non-Radioactive Cytotoxicity Assay (Promega) according to the manufacturer's instructions. All experiments were performed in triplicate wells and each experiment was repeated three times.

Statistical analyses of intracellular bacterial counts and host cell viability measurements were performed using Graphpad Prism v. 5.0c. Statistical significance between values from three or more groups was determined using one-way analysis of variance (ANOVA) with Tukey's post hoc test. Pairwise comparisons were performed using an unpaired Student's *t*-test, with Welch's correction where required. *p* < 0.05 is considered significant.

### (e) Fluorescent staining and video microscopy of *Salmonella* Typhimurium

Bacteria were stained with Alexa Fluor 594 carboxylic acid, succinimidyl ester (Invitrogen), as described by Turner *et al.* [24]. Staining with this dye has been previously observed to have no discernible effects on the motility of labelled bacterial cells [24]. RAW264.7 cells were seeded at a density of 2.5 × 10^5^ per dish in a 35 mm glass bottom cell-culture dish (Greiner Bio-One) in complete RAW medium and incubated for 24 h at 37°C, 5% CO_2_. The culture medium was removed and replaced with complete RAW medium without phenol red. The sample was mounted on the imaging stage of a Leica TCS SP5 microscope with a Leica DM6000 CFS confocal fixed stage system embedded in an environmental chamber (Leica Microsystems) at 37°C and the sample enclosed within a stage gas chamber; humidified 5% CO_2_ air was flowed through. Alexa Fluor 594 stained bacteria were added to the cells. Images were acquired through a 40× oil 1.25 NA objective, in the transmitted beam (bright field) and emission (fluorescence) channels, with 594 nm excitation every 753 ms for 30 min.

### (f) Image analysis for cell–cell contacts

Bacterium–cell contact numbers and durations were extracted from movies using a semi-automated approach. Three movies acquired on different days were analysed for each bacterial strain, except for *fliM*_P220L_ for which two movies were analysed. The number of macrophages and infection events in each movie was determined manually, assisted by a Matlab script for counting all bacterium–cell contacts and tracking the duration of each contact event. This analysis was performed on 400 frames (5 min) of each movie (see electronic supplementary material, table S3 for contact counts). Distribution of contact duration was obtained from this analysis of 400 frames. Infections are rare events and were determined by observation of the entire 30-min movie. In order to calculate percentage of infection per contact (PIC) event, the total number of bacterium–cell contact events over 30 min was estimated by assuming a constant rate of contacts and taking the 5 min value. Local MOI was extracted from each movie by dividing the average number of bacteria (over 400 frames) by the number of macrophages. The contact potential (*N*) of each bacterial stain was calculated as follows: *N* = *n*/(local MOI × *n*_wt_), where *n* is the average number of contact event per macrophage in 5 min, and *n*_wt_ is the number of contacts for the appropriate wild-type strain (SJW1103 for Δ*motAB* and Δ*fliOPQR*, and *fliM*_WT_ for *fliM*_R60C_ and *fliM*_P220L_) in 5 min, itself also normalized by its local MOI.

## 3. Results

### (a) Loss of flagellar motility by *Salmonella* Typhimurium reduces uptake by macrophages and this is further diminished by the presence of paralysed flagella

**Figure 1.**
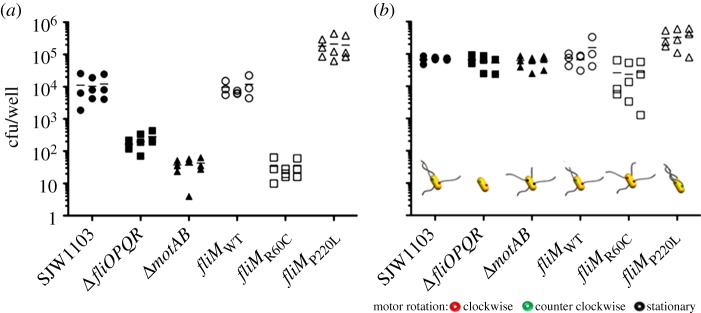
Intracellular bacterial counts from RAW264.7 macrophages infected with *S*. Typhimurium. Cells were infected with an MOI of 10 : 1 of the indicated strains as described in §2d. Data points show triplicate wells from three individual experiments. (*a*) The non-motile strains Δ*fliOPQR* and Δ*motAB* are taken up significantly less efficiently that the corresponding wild-type strain SJW1103. The tumble-biased *fliM*_R60C_ strain is internalized in significantly lower numbers than the corresponding wild-type *fliM*_WT_, whereas the run-biased *fliM*_P220L_ is taken up in significantly greater numbers. (*b*) The infection protocol was modified to include a centrifugation step as described in §2d. The centrifugation step increases the uptake of all strains, and equalizes the level of uptake of the non-motile strains Δ*fliOPQR* and Δ*motAB* to those of SJW1103 and *fliM*_WT_. Although it appears that uptake of *fliM*_R60C_ remains slightly lower than *fliM*_WT_, this apparent difference is not statistically significant. However, uptake of *fliM*_P220L_ is still significantly higher than *fliM*_WT_ despite centrifugation. Schematic of the motility phenotypes of each strain are shown below the counts. The cell body is shown in yellow, flagellar filaments in grey and flagellar motor in red (clockwise rotation), green (counterclockwise rotation) or black (stationary motor). (Online version in colour.)

In order to understand the role of flagellar motility in uptake of *S*. Typhimurium by macrophages, we generated a panel of mutants with a range of altered motility phenotypes. These strains are listed in the electronic supplementary material, table S1. The *fliOPQR* genes encode four of the six membrane bound components of the flagellar export apparatus and are required for flagellar assembly and motility [25,26]. Deletion of these components results in a strain that is non-motile and does not produce flagella. The *motA* and *motB* genes encode components of the flagellar motor that form the stator and proton channel which are anchored to the peptidoglycan layer, and deletion of these genes leads to a paralysed flagella phenotype, whereby the flagellar filament is assembled but unable to rotate [19,27–29]. These phenotypes were confirmed by video microscopy (electronic supplementary material, movies S1–S6). In order to determine whether motility, or the flagellum itself is important for uptake by RAW264.7 macrophages, we performed gentamicin protection assays using the SJW1103 wild-type strain, and Δ*fliOPQR* and Δ*motAB* mutants. Infections were performed with a MOI of 10 : 1 for 30 min, followed by gentamicin treatment for 1 h to remove extracellular bacteria. Macrophage viability was also measured in these experiments, and no significant differences between the survival of uninfected cells and those infected with any of the *S*. Typhimurium strains were detected (data not shown). This confirms that the observed differences in the intracellular viable counts do indeed reflect differences in bacterial uptake, rather than differences in the number of surviving macrophages. Both the Δ*fliOPQR* and Δ*motAB* strains are taken up by macrophages significantly less effectively than SJW1103 (*p* < 0.01), with uptake of these strains approximately 3 and 0.7% of wild-type levels (figure 1*a*). This suggests that motility, rather than the presence of the flagellum itself, is required for uptake of *S*. Typhimurium by macrophages. Internalization of the aflagellate Δ*fliOPQR* mutant is greater than the non-motile flagellated strain Δ*motAB* (*p* < 0.01), indicating that the presence of static flagella hinders uptake. A similar phenomenon has been observed with aflagellate and paralysed flagella mutants of *S*. Typhimurium with HEp-2 epithelial cells, with the authors postulating that the presence of flagella disrupts the necessary interactions between bacterium and host cell that allow internalization [17].

### (b) Centrifugation restores bacterial uptake of non-motile strains to wild-type levels

Centrifugation is a well-established method to enhance contact between bacteria and host cells in uptake experiments, and thereby mitigate for differences in motility [16–18]. We modified the gentamicin protection assay to include a 10 min centrifugation step at 500*g*, followed by static incubation for a further 20 min and then 1 h of gentamicin treatment. Centrifugation increases uptake of all strains, and equalizes the number of intracellular bacteria recovered following infection with SJW1103, Δ*fliOPQR* or Δ*motAB*, with no significant differences in the number of intracellular bacteria recovered between any of these strains (figure 1*b*). This suggests that contact between bacteria and macrophages is the primary factor determining internalization of *S.* Typhimurium, and that centrifugation can overcome defects that result from lack of motility, or the presence of static flagella. This differs from observations with *P. aeruginosa* and *V. cholerae*, where centrifugation does not fully restore uptake of non-motile mutants [18].

### (c) Bias toward a smooth-swimming phenotype dramatically increases uptake of *Salmonella* Typhimurium, whereas bias toward tumbling reduces uptake

In order to characterize how motility and cell contact influence uptake of *S*. Typhimurium by macrophages in more detail, infections were performed with strains expressing variant forms of the *fliM* gene. FliM forms part of the flagellar switch complex and mediates switching between clockwise and counterclockwise rotation states [30]. A large number of point mutations in *fliM* that bias flagellar rotation toward either the clockwise or counterclockwise mode have been described. Among these, point mutations that give rise to the R60C amino acid change result in a strong clockwise rotation bias and thus a predominantly tumbling phenotype. A P220L amino acid substitution produces a strong counterclockwise bias and, therefore, a smooth-swimming phenotype [30]. We used these mutations to investigate how the bias to tumbling or smooth-swimming influences uptake by macrophages. A *fliM* deletion mutant was transformed with plasmids carrying wild-type, R60C and P220L alleles of the *fliM* gene, hereafter referred to as *fliM*_WT_, *fliM*_R60C_ and *fliM*_P220L_, respectively. The wild-type, tumbling and smooth-swimming phenotypes of these strains were confirmed by microscopy (electronic supplementary material, movies S1–S6) and the strains were used to infect RAW264.7 macrophages (figure 1*a*). As expected, there were no significant differences between the levels of uptake by macrophages of *S*. Typhimurium wild-type with *fliM*_WT_ *in trans* and the SJW1103 parent strain alone. The *S*. Typhimurium *fliM*_R60C_ strain is taken up by macrophages much less efficiently than *fliM*_WT_ (*p* < 0.01), with recovered intracellular bacteria reaching only approximately 0.3% of wild-type levels. Conversely, the *fliM*_P220L_ strain is taken up by macrophages in significantly greater numbers than *fliM*_WT_ (*p* < 0.05), with 20.8-fold more intracellular bacteria recovered. The enhanced uptake of the run-biased *fliM*_P220L_ strain suggests that this mode of motility promotes uptake by macrophages, whereas *fliM*_WT_, which exhibits a combination of both modes of motility, has a level of uptake that falls between *fliM*_R60C_ nd *fliM*_P220L_.

### (d) Centrifugation does not fully equalize the uptake of smooth-swimming *Salmonella* Typhimurium to wild-type levels

Macrophage uptake assays were then performed on these strains with the inclusion of the centrifugation step (figure 1*b*). As observed with the wild-type and non-motile strains, centrifugation increases uptake of the *fliM*_WT_, *fliM*_R60C_ and *fliM*_P220L_ strains. Centrifugation restores uptake of *fliM*_R60C_ to a level that is not significantly different from the wild-type. However, uptake of centrifuged *fliM*_P220L_ remains significantly higher (*p* < 0.01) than *fliM*_WT_ with approximately 3.4-fold more intracellular bacteria recovered. This indicates that although the contact between bacteria and macrophages is critical in influencing the uptake of these strains, it cannot fully explain the behaviour of the run-biased strain. Despite enhanced contact by centrifugation, uptake of *fliM*_P220L_ remains higher than the wild-type. Therefore, some feature of the interaction between the macrophage and bacterium is altered by the balance between the run and tumble modes of motility, and we demonstrate that contact duration is a key factor influencing uptake.

### (e) All mutant salmonellae have less contact initiation with macrophages than wild-type strains

In order to investigate how contact between *S.* Typhimurium and RAW264.7 macrophages influences invasion, we set up a system to observe interactions between the bacteria and host cells directly. Bacteria were stained with Alexa Fluor 594 carboxylic acid, succinimidyl ester dye, which stains both the cell body and flagellar filaments [24]. These bacteria were then used to infect the macrophages at a MOI of 10 and imaged every 753 ms for 30 min. To quantify the potential of each strain to initiate contact with a macrophage, it is necessary to know the number of macrophages and bacteria that are present, and to enumerate all individual contact events. When macrophage cultures were inoculated with a *Salmonella* suspension to give a calculated MOI of 10, bacteria were distributed throughout the volume of the inoculum, but only a proportion of the bacteria were near the macrophage monolayer. In order to quantify any differences between strains in their distribution throughout the tissue culture media due to differences in the motility phenotype a ‘local MOI’ was calculated from each movie.

**Figure 2.**
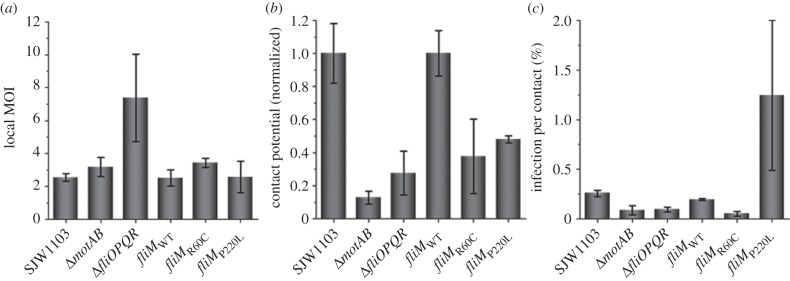
Bacterial motility alters the contact potential of *S*. Typhimurium and frequency of infection upon contact with macrophages. The ‘local MOI’ (defined in §2d) was calculated for each strain (*a*) and used to determine the normalized average number of contact events per macrophage initiated by each strain (*b*). The ‘contact potential’ (defined in §2f) is similar between the tumble-biased and the run-biased strains. These two biased strains, however, initiate about half as many contact events than the wild-type strains and at least twice as many than the non-motile strains. Upon contact, non-motile and tumble-biased strains infect significantly less frequently than the wild-type upon contact with macrophages, while the run-biased strain infects about four times more than the wild-type strains upon contact (*c*). Error bars show the standard error of the mean.

The local MOIs are similar for all strains except for the aflagellate strain (Δ*fliOPQR*) (figure 2*a*). We propose that this difference is because in the absence of flagella, the bacteria sediment more rapidly than in the presence of flagella, causing an increase in the local MOI (electronic supplementary material, figure S2). We used the respective local MOI values to assess the ability of each strain to initiate contact with the macrophages. For each strain, the number of contact events per macrophage was divided by the local MOI and normalized against the appropriate wild-type strain. The resulting calculations show that non-motile and tumble-biased strains are significantly less able to initiate contact with macrophages than wild-type strains (figure 2*b*). Somewhat surprisingly, the run-biased strain (*fliM*_P220L_) also shows similar contact potential to the tumble-biased strain (*fliM*_R60C_).

### (f) Non-motile and tumble-biased strains infect less frequently while the run-biased strain infects more frequently upon macrophage contact

The number of infection events and bacterium–cell contact events were extracted from each movie (three movies per strain, see electronic supplementary material, figure S1 for an image of bacterium–macrophage contacts) and used to calculate the average PIC (the number of infection events per 100 contacts) for each bacterial strain (figure 2*c*). PIC values were very low (0.05% to 1.25%), confirming our previous observation that infection events are rare upon contact [20]. Wild-type strains (SJW1103 and *fliM*_WT_) have similar PICs of 0.26 ± 0.03% and 0.20 ± 0.01% respectively. The non-motile strains Δ*motAB* and Δ*fliOPQR*, respectively, infect 2.9 and 2.7 times less than SJW1103 upon contact, with PICs of 0.09 ± 0.05% and 0.10 ± 0.01%. The tumble-biased *fliM*_R60C_ strain infects 3.7 times less frequently than the wild-type (*fliM*_WT_) with a PIC of 0.05 ± 0.02%_._ The most striking difference in PIC was found in the run-biased strain *fliM*_P220L_, which infects 6.3 times more than the wild-type strain (*fliM*_WT_) with a PIC of 1.25 ± 0.75%. This trend is consistent with the population-level measurements obtained from the gentamicin protection assays with these strains (figure 1*a*).

### (h) The run-biased strain maintains longer contact with the macrophage than wild-type strains

**Figure 3.**
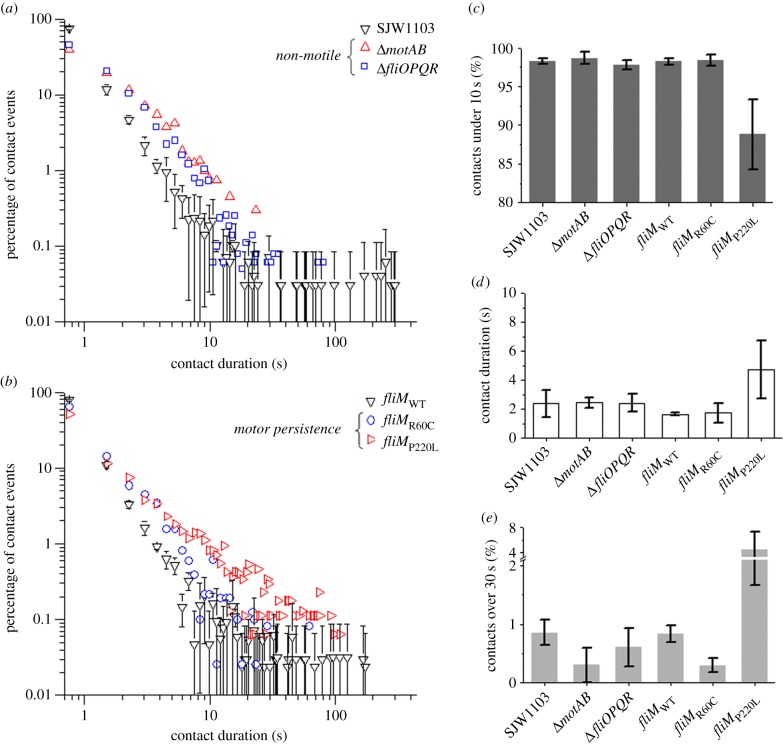
Distribution of bacterium–macrophage contact durations. The run-biased strain initiates longer contacts than the other strains. Single contact events between *S*. Typhimurium bacteria and RAW264.7 macrophages were counted and timed. The data in (*a*) and (*b*) show the distribution of the duration of contact events counted for each bacterial strain (error bars are the standard deviation). (*c*) Proportions of contact events that last under 10 s reveal that approximately 98% of contacts last less than 10 s for all strains except for the run-biased *fliM*_P220L_ strain for which this proportion is around 88%. (*d*) The average contact duration is similar between the wild-type, tumble-biased and non-motile strains. (*e*) Proportions of contact events that last over 30 s are lower in non-motile and tumble-biased strains and higher in the run-biased strain compared with the wild-type strains. Error bars show the standard error of the mean. (Online version in colour.)

The distribution of bacterium–macrophage contact duration was obtained from our time-lapse imaging for each bacterial strain (figure 3*a*,*b*). These distributions confirmed our previous observation that the majority of contact events last less than 10 s (figure 3*c*) [20]. However, the proportion of contact events that are less than 10 s differs between strains. Non-motile strains (Δ*motAB* and Δ*fliOPQR*) and the tumble-biased strain (*fliM*_R60C_) show, respectively, 98.7 ± 0.7%, 97.7 ± 0.6% and 98.4 ± 0.7% of events under 10 s. These values are similar to the wild-type strain values (SJW1103 and *fliM*_WT_) of 98.3 ± 0.4% and 98.2 ± 0.4%. This proportion is much lower for the run-biased strain (*fliM*_P220L_) at 88.8 ± 4.5%. Average contact durations were similar between the non-motile strains Δ*motAB* and Δ*fliOPQR* (2.4 ± 0.3 s and 2.4 ± 0.6 s, respectively), and the wild-type strain SJW1103 (2.4 ± 0.9 s). The tumble-biased strain *fliM*_R60C_ also shows an average contact duration that is very similar to its corresponding wild-type strain *fliM*_WT_ (1.7 ± 0.7 against 1.7 ± 0.1 s). The run-biased strain *fliM*_P220L_ has an average contact duration 2.8 times longer compared with the wild-type strain *fliM*_WT_ (4.7 ± 2.0 against 1.7 ± 0.1 s) (figure 3*d*).

## 4. Discussion

Consistent with previous reports, our data show that when measured at a population-level, *Salmonella* strains with paralysed flagella, lacking flagella, or strains that are biased towards tumbling show a defect in their ability to infect host cells, whereas run-biased strains exhibit increased uptake [16,17]. Our previous work [20] showed that very few wild-type *S*. Typhimurium–macrophage contact events result in cellular infection. Non-motile and tumble-biased bacterial strains have been suggested to be less infectious because of their impaired ability to initiate contact with cells when compared with wild-type strains [16,17]. The rescue of the uptake levels of non-motile strains by centrifugation to the levels of infection seen with wild-type bacterial strains supports this concept (figure 1) [16].

The PIC was calculated for each of the bacterial strains. PICs were low for all strains (less than 1.3%) as predicted by our models [20]. There were significant differences between strains with different motility phenotypes (figure 2*c*). Non-motile strains infect significantly less (approx. threefold less) and the run-biased strain infects significantly more (greater than sixfold) than wild-type strains upon macrophage contact. These results suggest that the differences in bacterial uptake between strains of differing motility cannot be explained simply by the number of bacterium–cell collisions. Our previous work showed that most bacterium–cell contact events are very short, with 95% lasting under 10 s, and that contact events longer than 10 s have a high probability of resulting in successful cellular infection [20]. We timed the duration of infection events from the moment bacterium–cell contact was initiated to complete internalization of wild-type *S.* Typhimurium in macrophages. The average duration of contact to infection was 2.2 ± 0.2 min, with the shortest recorded event lasting 27 s (see electronic supplementary material, figure S3). The distribution of contact duration for each bacterial strain confirmed our previous observation that most contact events last less than 10 s; these short periods of bacterium–cell contact represented about 98% of all contact events for all strains, except for the run-biased strain for which only 88% of all contact events lasted less than 10 s (figure 3*c*). Our results also show that non-motile bacteria, once in contact with the macrophage, had similar average periods of time associated with the cell as wild-type strains, while the run-biased strain maintained the longest bacterium–cell contact time (figure 3*d*). These results suggest that bias towards the run phase of bacterial motility promotes macrophage infection by increasing duration of the bacterium–cell contact time.

Non-motile *Salmonella* strains have similar numbers of short contact events (under 10 s, figure 3*c*) and similar average contact durations (figure 3*d*) to the corresponding wild-type strains. This suggests that average contact time alone cannot account for the differences in levels of uptake observed between wild-type and non-motile strains. The contact duration distribution curves can be divided into two sections: (i) a power-law decay lasting up to between 10 and 30 s, and (ii) a plateau composed of sporadic long events that reach out to 5 min (figure 3*a*,*b*) and are anomalous in comparison to the regime (i). Interestingly, there are differences in the plateau phase between strains. We have measured the shortest adhesion time successfully leading to internalization as 27 s, a duration that corresponds closely to the transition between power law and plateau. A comparison of the proportion of contact events that last over 30 s between the bacterial strains (figure 3*e*) reveals that they correlate strongly with the PIC values (figure 3*c*). Our observations, therefore, suggest that macrophage infection by *S*. Typhimurium is influenced both by contact frequency and contact duration, with a higher frequency and longer duration of contact favouring infection. The run-biased strain infects particularly efficiently, most likely because the extended contact duration between the bacterium and host cell allows more time for uptake, primarily via macropinocytosis [8,31]. It has been suggested that macrophages are able to sense flagellar rotation directly to initiate the infection process, but the mechanism by which this may occur is unclear. This conclusion is largely based on the observation that progressive loss of bacterial motility correlates with decreasing macrophage infection by *P. aeruginosa* and *V. cholerae* [18]. It would be interesting to investigate the contact frequency and duration of these bacteria to see whether the stepwise reductions in motility also reduce these parameters.

Our data show that contact time is a critical factor in facilitating bacterial infection of macrophages, but bacterial shape may also influence bacterial uptake rate. Our previous work [32] suggests the importance of local curvature, since elliptical particles are taken up more slowly than spherical particles. Motility may therefore also enhance uptake of rod shaped bacteria such as *Salmonella*, by biasing the initial contact to occur pole-first, as shown in electronic supplementary material, figure S1*a*.

Predictably, non-motile strains are less able to initiate contact than the wild-type (figure 2*b*). However, although the run-biased strain has greater contact potential than non-motile strains, it unexpectedly has a reduced potential for contact initiation than the wild-type. This potential also appears to be close to that of the tumble-biased strain. To explain this it is necessary to consider that the run-biased strain tends to maintain longer contact events (figure 3*d*) as well as to infect more upon contact compared with wild-type strains (figure 2*c*). This results in a significant decrease in the number of ‘free’ bacteria available to initiate new contacts over time.

## 5. Conclusion

We have shown that the balance between the tumble and run phases of motility in *Salmonella* critically influences how efficiently bacteria can infect macrophages. Although contact frequency is a key parameter effecting infection efficiency, the average durations of those contact events (the vast majority of which are transient) are also important. Bacterium–cell adhesion in excess of 10 s is required to initiate macrophage infection, and extended contact times are favoured by the run phase of motility. These results suggest that some part of the complex combination of molecular (ligand/receptor signalling) and physical (membrane engulfment) events that drive bacterial infection of macrophages requires a minimum duration on this timescale—the details of this mechanism remain to be established.

## Supplementary Material

Supplementary materials
