# Supplementary material for: The frequency and duration of Salmonella–macrophage adhesion events determines infection efficiency
Source: Philos Trans R Soc Lond B Biol Sci. 2015 Feb 5;370(1661):20140033. doi: 10.1098/rstb.2014.0033 (PMC4275903; doi:10.1098/rstb.2014.0033)
Supplement: Supplementary materials [file rstb20140033supp1.pdf]

Achouri, S. *et al*. The frequency and duration of *Salmonella*-macrophage adhesion events determines infection efficiency. *Phil. Trans. R. Soc. B.* doi: 10.1098/rstb.2014.0033

SUPPLEMENTARY MATERIALS

Supplementary Table 1. Bacterial strains and plasmids used in this study.

**Strain**	**Characteristics**	**Source/Reference**
S.Typhimurium SJW1103	Wild-type, phase-1 locked, motile strain	[33]
S.Typhimurium Δ*fliOPQR*	SJW1103 derivative, Δ*fliOPQR*	[21]
S.Typhimurium Δ*motAB*	SJW1103 derivative, Δ*motAB*	This study
S.Typhimurium Δ*prgH*	SJW1103 derivative, Δ*prgH*	This study
S.Typhimurium Δ*fliM*	SJW1103 derivative, Δ*fliM*	This study
**Plasmid**	**Characteristics**	**Source/Reference**
pACTrc	pTrc promoter, p15A origin of replication; lacIq, CmR	[22, 34]
pACTrc-$fliM_{WT}fliN_{WT}$	Complements motility of *S*.Typhimurium ΔfliM	This study
pACTrc-$fliMR_{60C}fliN_{WT}$	Tumbling frequency increased	This study
pACTrc-$fliM_{P220L}fliN_{WT}$	Tumbling frequency decreased	This study
pKD46	pINT-ts derivative. araB promoter Express Red recombinase, γ, β and exo genes of phage λ, Temperature-sensitive origin of replication, ApR	[35]
pKD13	pANTSγ derivative, template plasmid harboring kanamycin resistance marker flanked by FRT sites, ApR, KmR	[35]

Supplementary Table 2. Oligonucleotide primers used in this study.

Oligonucleotide	Sequence 5' – 3'
fliMFOR	GCTCCTGATCCTCTAGAAGGAGAAACGATATGGGCGATAGTATTCTTTCT
fliNREV	CGAGTCTCTAGATCATCATTAACGACTCAAACGACGC
fliMR60CFOR	GATTATTAACGAGTGCTTCGCACGCCAGT
fliMR60CREV	GAACTGGCGTGCGAAGCACTCGTTAATAAT
fliMP220LFOR	GAATTTAACATCTGTCTGCTGTTTAGCATGAT
fliMP220LREV	CTCGATCATGCTAAACAGCAGACAGATGTT
motAdelFOR	CTGCGCATCCTGTCATAGTCAACAGCGGAAGGATGATGTCGTGCTTATCTTATTA GGTTACATTCCGGGGATCCGTCGACC
motBdelREV	AAAATCGCTAATATCCATGCTCACGCTATCACCTCGGTTCCGCTTTTGGCGATGT GGGTGTAGGCTGGAGCTGCTTCG
prgHdelFOR	ATCTGCTGCTATCGAGAACGACAGACATCGCTAACAGTATATATGGAAACATCA AAAGAGATTCCGGGGATCCGTCGACC
prgHdelREV	CTGACCAAGGTGTTGCCATAATGACTTCCTTATTTACGTTAAATTAAAGTGGGCT TGGGAATGTAGGCTGGAGCTGCTTCG
fliMdelFOR	CTGACGTGCTTTATACAGCTTTTATTCTGCGGTAACGATATGGGCGATAGTATTC TTTCTATTCCGGGGATCCGTCGACC
fliMdelREV	CGCCAGTATTCTCATCGGACGGATTATTCATGTCACTCATTTGGGCTGTTCCTCAT TCAGTGTAGGCTGGAGCTGCTTCG

Supplementary Table 3. Six bacterial strains were used to investigate the role of *S.* Typhimurium's motility behaviours in its infection of macrophages. *S.* Typhimurium's motility is based on the synchronicity of its flagella's rotation. Its normal motility phenotype is bimodal, consisting of: runs when all flagella rotate counterclockwise, and tumbles when one or more of the flagella rotate clockwise. Two of these bacterial strains were used to characterise the wild-type behaviour, and the other four strains were selected to each trigger a specific feature or *S.* Typhimurium's motility machinery.

**Strain**	**Motility phenotype**	**Number of contacts counted**
SJW1103	Motile: Wild type	2962
FliM_{WT}	Motile: Wild type	3556
Δ*motAB*	Non-motile: Stationary flagellar motors	331
Δ*fliOPQR*	Non-motile: Aflagelate	1633
$\textit{fliM}_{\text{R60C}}$	Non-motile: Tumble-biased	1792
$\text{fliM}_{\text{P220L}}$	Motile: Run-biased	1221

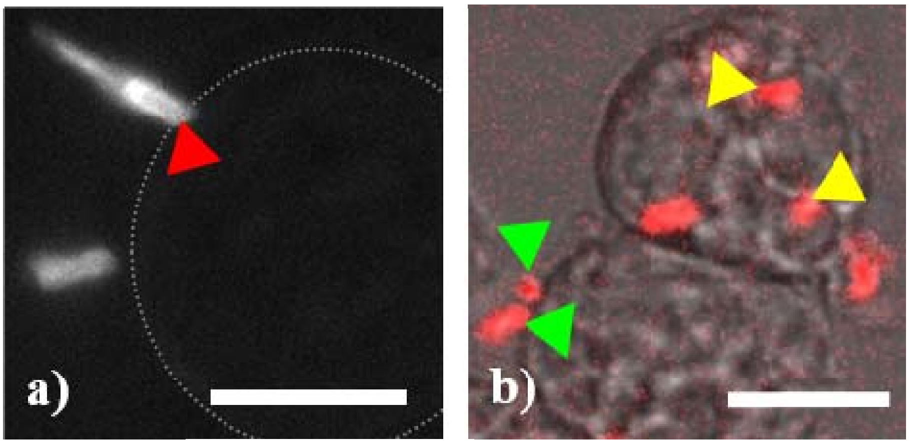

Supplementary Figure 1: Live fluorescence imaging of bacterial-cell contact dynamics. (a) *S.* Typhimurium bacterium of the SJW1103 strain stained with Alexa FLUOR 594 in contact with a macrophage (edges indicated by the white dotted circle). The red arrow points at the contact point between the bacterium and the macrophage revealing a “pole-first” contact (the scale bar corresponds to 5µm). (**b)** Confocal image of macrophages infected with *S.* Typhimurium SJW1103 stained with Alexa Fluor 594 (red). Yellow arrows show bacteria taken up by macrophages. Green arrows show bacteria in transient contact with macrophages (the scale bar corresponds to 10µm).

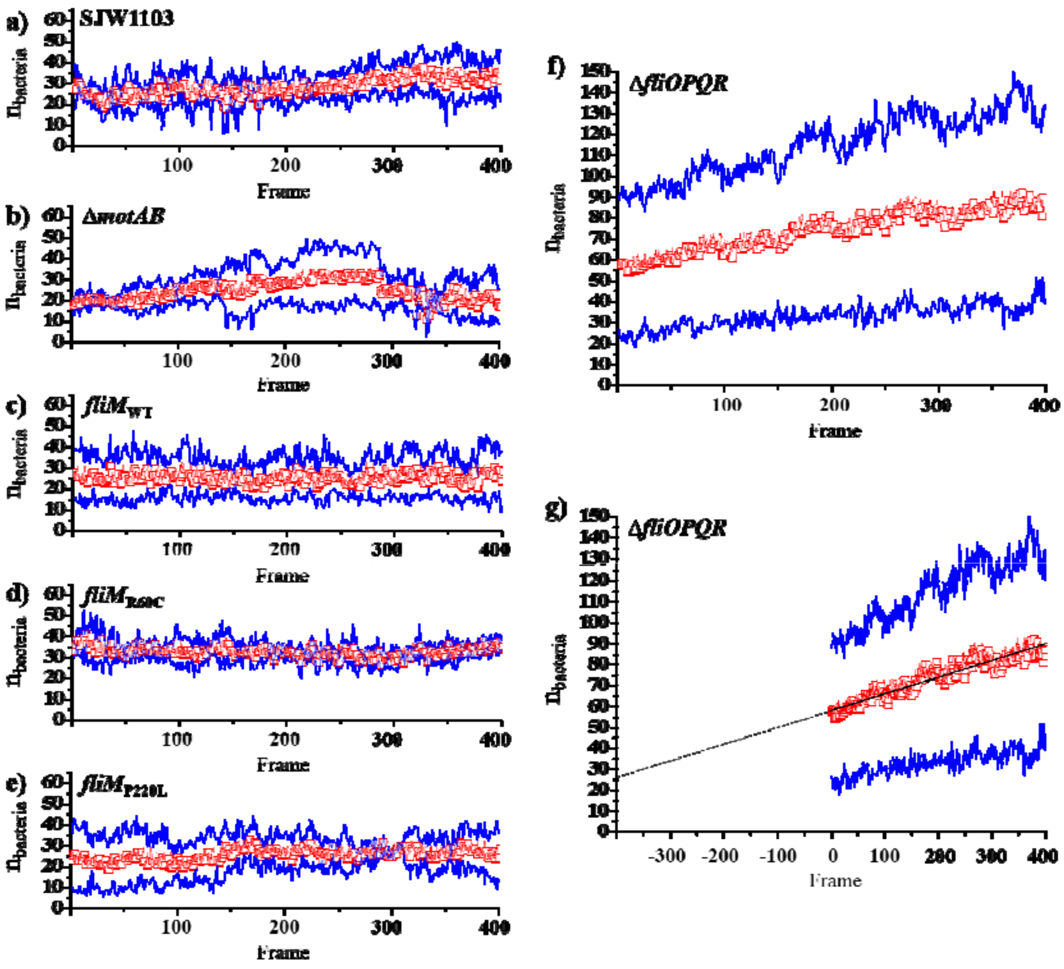

Supplementary Figure 2: Local MOIs over time. The aflagellate strain Δ*fliOPQR* sediments fast causing an increase in local MOI. For each bacterial strain, the number of bacteria ($n_{bacteria}$) was detected in each frame using a Matlab script. This number – average $n_{bacteria}$ per frame (red) averaged between 3 movies per strain with standard deviation (blue) – was relatively stable and similar for all bacterial strain (a-e) except for the aflagellate strain (f) for which the number of bacteria detected per frame increased steadily throughout the movies (400 frames). There was a delay of roughly 5 minutes between the moment the bacterial inoculum was added to the sample and the moment the acquisition was started (due to adjustment of sample, environmental chamber, and imaging settings) Using a linear fit, we predict that when the inoculum was added, the local MOI for the aflagellate strain was close to the ones found for the other strains.

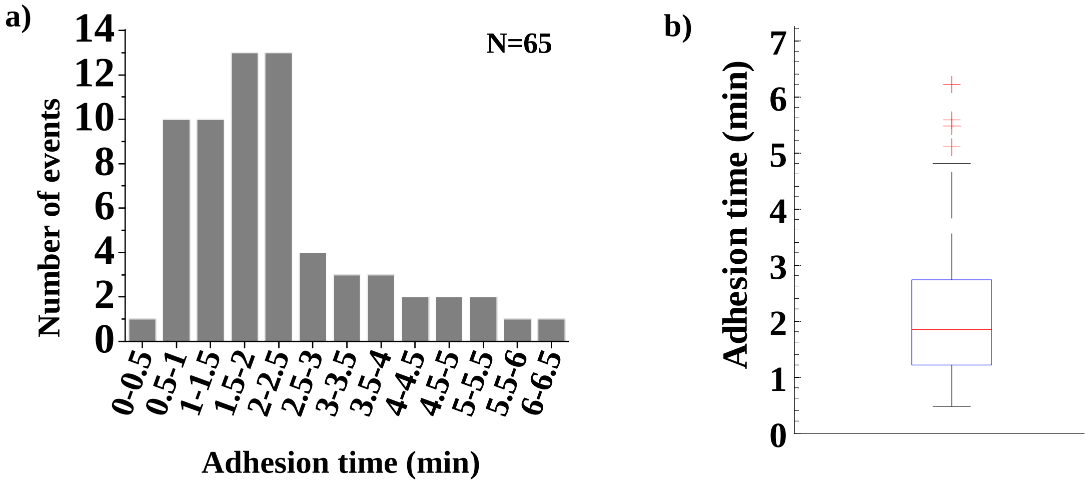

Supplementary Figure 3: Adhesion duration before infection. RAW 264.7 macrophages were inoculated with a suspension of *S.* Typhimurium (JH3016 strain, wild-type motility phenotype). The adhesion time before infection was measured from initial bacterial-cell contact to complete internalization for 65 infection events. A distribution of these adhesion times is shown in a) and a box plot in b).

Descriptions of other supplementary files

Supplementary movie 1: Fluorescence microscopy movie of SJW1103 *salmonella* bacteria labeled with Alexa Fluor® 594. This strain exerts a wild-type motility phenotype with runs (directional displacement) and tumbles (erratic movement).

Supplementary movie 2: Fluorescence microscopy movie of Δ*motAB salmonella* bacteria labeled with Alexa Fluor® 594. This strain is non-motile, its flagellar motors are stationary.

Supplementary movie 3: Fluorescence microscopy movie of Δ*fliOPQR salmonella* bacteria labeled with Alexa Fluor® 594. This strain is non-motile, it does not possess any flagella.

Supplementary movie 4: Fluorescence microscopy movie of $fliM_{\mathrm{WT}}$ *salmonella* bacteria labeled with Alexa Fluor® 594. This strain exerts a wild-type motility phenotype with runs (directional displacement) and tumbles (erratic movement).

Supplementary movie 5: Fluorescence microscopy movie of $fliM_{\mathrm{R60C}}$ *salmonella* bacteria labeled with Alexa Fluor® 594. This strain exerts a motility phenotype biased towards tumbles.

Supplementary movie 6: Fluorescence microscopy movie of $fliM_{\mathrm{P220L}}$ *salmonella* bacteria labeled with Alexa Fluor® 594. This strain exerts a motility phenotype biased towards runs.
